# Community versus institutionalised care for people with severe mental illness in five countries in Southeast Europe: pooled analysis of five randomised trials

**DOI:** 10.1136/bmjgh-2024-018594

**Published:** 2025-10-23

**Authors:** Laura Shields-Zeeman, Filip Smit, Ben Wijnen, Catharina Roth, Michel Wensing, Ionela Petrea, on behalf of the RECOVER-E consortium, Felix Bolinski, Felix Bolinski, Stojan Bajraktarov, Jovo Dedovic, René Keet, Martina Rojnic Kuzman, Vladimir Nakov, Raluca Nica, Antoni Novotni, Aleksandar Tomcuk, Tatjana Djurisic, Guadalupe Morales, Tiberiu Rotaru Anghelescu

**Affiliations:** 1 Utrecht University, Utrecht, The Netherlands; 2 Trimbos Institute, Utrecht, The Netherlands; 3 Department of Biostatistics and Epidemiology and Department of Clinical, Neuro and Developmental Psychology, Amsterdam Public Health Research Institute, VU University Medical Center, Amsterdam, The Netherlands; 4 Department of General Practice and Health Service Research, University Hospital Heidelberg, Heidelberg, Germany; 5 INSIGHT International, London, UK; 6 University Clinic of Psychiatry, Skopje, North Macedonia; 7 Special Psychiatric Hospital Dobrota, Kotor, Montenegro; 8 GGZ Noord-Holland-Noord FIT-Academy, Heerhugowaard, The Netherlands; 9 University of Zagreb School of Medicine, Zagreb, Croatia; 10 National Center of Public Health and Analyses, Sofia, Bulgaria; 11 Romanian League for Mental Health, Bucharest, Romania; 12 Institute of Public Health of Montenegro, Podgorica, Montenegro; 13 Fundación Mundo Bipolar, Madrid, Spain; 14 Siret Psychiatric Hospital, Siret, Romania

**Keywords:** Mental Health & Psychiatry, Randomised control trial, Health services research

## Abstract

**Background:**

The RECOVER-E project implemented community-based mental healthcare (CMH) oriented at functional recovery in people with schizophrenia, bipolar and severe major depressive disorder in five countries: Bulgaria, Croatia, Montenegro, North Macedonia and Romania, with the aim to shift care from institutions to communities.

**Objective:**

To evaluate the effectiveness of CMH under real-world circumstances across various healthcare ecologies and contexts.

**Methods:**

A randomised comparison of CMH versus treatment as usual (TAU) based on pooled data from all five RECOVER-E trials (N=931). Outcomes were personal and social role functioning (WHO Disability Assessment Schedule, WHODAS 2.0) and health-related quality of life (EuroQoL-5 Dimensions-3 Levels) at baseline, 12 and 18 months postbaseline. Intention-to-treat analysis was conducted with mixed modelling and a sensitivity analysis adjusted for the impact of COVID-19 on healthcare delivery and outcomes.

**Findings:**

At 18-month follow-up, CMH had a 4.55 lower WHODAS disability score than TAU, which was significant (b=−4.55, SE=1.21, z=−3.75, p<0.001), and improved quality of life by 0.07 utility (b=0.07, SE=0.014, z=4.56, p<0.001) equivalent to an additional 25 days in full health. Similar effects were observed in each of the five countries and for all WHODAS subdomains (cognition, mobility, self-care, getting along with people, life activities, participation). Sensitivity analyses adjusting for the confounding effect of COVID-19 showed similar effects.

**Clinical implications:**

Recovery-oriented CMH for people with severe mental illness was effective in improving functioning and quality of life for people with schizophrenia, bipolar disorder and severe depression in five South-Eastern European countries and could be implemented across different health systems.

**Trial registration numbers:**

Bulgaria: NCT03922425, Croatia: NCT03862209, Macedonia: NCT03892473, Montenegro: NCT03837340, Romania: NCT03884933.

**WHAT IS ALREADY KNOWN ON THIS TOPIC**

- The prevailing institutional care model in Southeast European countries often does not produce good clinical outcomes or enhance quality of life.
- Community mental healthcare (CMH) offers promising benefits over institutionalised care, for example, on clinical and social outcomes, but evidence of its effectiveness, particularly in Southeast Europe, is limited.

**WHAT THIS STUDY ADDS**

- Clinical evidence from five trials in five countries on the effectiveness of locally adapted CMH and their effective implementation in different health system ecologies.

**HOW THIS STUDY MIGHT AFFECT RESEARCH, PRACTICE OR POLICY**

- These findings may drive further transformation towards CMH in Southeast Europe and other low-resource settings.
- Findings indicate that tailoring intervention content and implementation strategies to local contexts is crucial for effective implementation.

## Background

 The RECOVER-E project aimed to develop, implement and evaluate recovery-oriented community mental healthcare (CMH)^1^ for people with schizophrenia, bipolar disorder and severe depressive disorder across five sites in Southeast Europe (SEE): Bulgaria, Croatia, Montenegro, North Macedonia and Romania. It was designed to support the transition from essentially institutionalised mental healthcare towards evidence-based and recovery-oriented CMH.

Historically, psychiatry in parts of SEE has been associated with stigmatising attitudes and discriminating practices; conditions in some psychiatric hospitals remain inadequate, sometimes inhumane and the treatment of people with mental health problems is still marked by paternalistic practices.^2 3^ Additionally, psychiatrists working in SEE still prefer passive clinical decision-making rather than shared and active decision-making styles in their everyday practices.^4^ From the 1990s onwards, many countries in the SEE region started to transform their healthcare systems.^5^ Several have made efforts to transition care towards CMH, that is, deinstitutionalisation.^6^ The RECOVER-E project aimed to contribute to this process in the five SEE countries.

The project entails a complex intervention,^7^ as it comprises multiple components and involves the collaboration of many stakeholders at different levels under real-world circumstances to engender the intended healthcare system transformation across five different but largely under-resourced mental health ecosystems. Due to this complexity, the broader research aim was to deliver ‘actionable evidence’ for transforming traditional mental health systems towards evidence-based CMH practices by ascertaining that CMH is (1) acceptable, (2) implementable, (3) effective, (4) cost-effective and (5) transferable across settings and contexts. In the current paper, we focus on the evaluation of the effectiveness of CMH under real-world circumstances and its implementation across various health system ecologies and contexts.

The current effectiveness evaluation of CMH vis-à-vis treatment as usual (TAU) is based on the pooled data from five pragmatic randomised controlled trials (RCTs) conducted in the five sites. We tested the following hypotheses: compared with traditional TAU, (1) participants receiving CMH will show better personal and social role functioning; (2) CMH will yield a better treatment response defined as a clinically relevant improvement in functioning, (3) CMH will generate better health-related quality of life. Finally, (4) when all five sites show similar patterns of improvement in the participants’ personal and social functioning, then we will take that as a pointer for the apparently successful implementation of CMH services across different healthcare ecologies and contexts.

## Methods

### Study design

Five two-arm non-blinded pragmatic RCTs were conducted in five SEE sites: Kotor in Montenegro, Skopje in North Macedonia, Sofia in Bulgaria, Siret in Romania and Zagreb in Croatia. Consenting participants were individually randomised to receive either CMH or TAU. Assessments were conducted at baseline (t_1_) with follow-ups at 12 and 18 months postbaseline (t_12_ and t_18_). Detailed information has been published in the study protocol.^8^ The reporting of this study complies with the Consolidated Standards of Reporting Trials (CONSORT) guidelines for RCTs^9^ and for reporting outcomes.^10^ A populated CONSORT and TIDieR checklist for reporting of interventions^11^ is added (onlinesupplemental materials 1  2).

### Participants

Participants were service users aged 18 years and older meeting the diagnostic criteria for bipolar disorder, severe major depression, schizophrenia, schizophreniform or schizoaffective disorder according to the 10th revision of the International Statistical Classification of Diseases and Related Health Problems^12^ not in symptomatic remission and in need of continued care; and having severe limitations in personal and social role functioning (as per the International Classification of Functioning, Disability and Health, ICF^13^). All participants had to provide written informed consent and were excluded from the trial if they had dementia or other severe medical conditions or were receiving mandated treatment.

Participants were recruited into the trial in three steps, with each subsequent recruitment step being applied when the previous step did not yield the targeted sample size. The first step was directed at people meeting inclusion criteria and making a first entry into the mental healthcare system (ie, first admissions without a prior treatment history). The second step was directed at service users with a prior treatment history of accessing mental healthcare but currently making a re-entry into the mental healthcare system (ie, readmissions or service users starting treatment for a new episode). The third step included service users who were receiving care for less than a year. Recruitment of participants into the trial, including eligibility assessment, was led by the psychiatrists working at the hospital in each of the sites.

### Patient and public involvement

We conducted a comprehensive needs assessment in all sites before data collection to ensure that the project aligned with local needs, policy priorities and resources. Moreover, we fostered public involvement by placing responsibility, for example, regarding the design of the study, as well as recruitment and clinical work, with the local research leads in all sites. Lastly, though service users were not directly involved in the research process in each of the five sites, a peer expert (GM) was crucial in developing and providing training for service providers in the multidisciplinary community mental health teams, and for training persons with lived experience as peer workers, members of the CMH team. A detailed reflexivity statement is provided as online supplemental material 3.

### Randomisation and masking

Individual simple randomisation (1:1 allocation) using random.org for obtaining true random numbers was carried out in a concealed way by an independent statistician at each of the five sites. Masking of clinicians and participants for the intervention was not feasible. The analysts who conducted the effect evaluation were not masked for the participants’ randomisation status.

### Community-based mental healthcare

CMH was delivered by newly introduced multidisciplinary CMH teams. Each team had at least one nurse, psychiatrist, psychologist, social worker and a peer worker (person with lived/living experience of a severe mental illness). The CMH teams consisted of hospital staff allocated to work on these teams. Each team was trained to deliver care according to an adapted version of the Flexible Assertive Community Treatment (F-ACT) service delivery model.^14 15^ Members of teams were trained to focus on recovery goals of service users in a strengths-based approach to care,^16^ as well as timely and appropriate care in the event of a crisis.^8 17^ The F-ACT blueprint of workflows, staff requirements and logistics (technology, transport to carry out home visits) was adapted to each site’s resources with the systematic involvement of local stakeholders. Consultations by the CMH team with service users were primarily through home visits or outpatient consultations elsewhere in the community. Services delivered by the teams included pharmacological treatment, psychological treatment and referral to social care. The teams had a shared caseload and had regular meetings together to assess the status and needs of service users allocated to the team, to determine who needed more intensive case management and who did not. At the start of the RECOVER-E project, all members of the CMH teams received 2 weeks of face-to-face training on working in a multidisciplinary CMH team and discipline-specific training such as working as a peer worker. Refresher training sessions were provided online (due to the COVID-19 pandemic) through assessment of the needs of the CMH team members in the five sites once in every month or 2 months. More information about the overall implementation of CMH across the five sites can be found in the Template for Intervention Description and Replication (TIDieR) checklist for reporting interventions (see online supplemental material 2).

### Treatment as usual

TAU consisted of the existing mental health service provision as offered at each site, which delivered treatment either in the inpatient or outpatient department of a hospital. Most often, TAU consisted of pharmacological treatment and limited psychological treatment. At the start of the project, all sites stated that none of their hospitals had specialised CMH teams. An overview of usual care for people with SMI in each of the five sites in the five countries is described elsewhere.^8^

### Outcomes

All outcomes were assessed through self-report, administered to participants by designated research teams, who were coordinated by the research site lead (country-level principal investigator). The primary outcome was (reductions in) disability in personal and social role functioning as assessed by the 36-item version of the WHO Disability Assessment Schedule, WHODAS 2.0.^18^ WHODAS measures functional disability according to the ICF.^18^ Six domains of functioning were assessed: Cognition (understanding and communicating), Mobility (moving and getting around), Self-care (hygiene, dressing, eating and staying alone), Getting Along (interacting with other people), Life Activities (domestic responsibilities, leisure, work and school) and Participation (joining in community activities). WHODAS 2.0 has a stable factor structure, high internal reliability (Cronbach’s α=0.86) and high test–retest reliability (intraclass correlation ρ=0.98).^19^ WHODAS scores range from 0 (no disability) to 100 (full disability).

To facilitate clinical interpretation, WHODAS was converted into treatment response, defined as a clinically relevant improvement at individual level and occurs when a participant improves >6 points on the WHODAS from baseline to last follow-up. This corresponds to a standardised mean change of d>0.33, for which this trial was powered.^20^

The EuroQoL with 5-Dimensions and 3-Levels (EQ-5D-3L) was used to measure health-related quality of life. The EQ-5D-3L is a widely used quality of life instrument and contains five dimensions (mobility, self-care, daily activities, pain/discomfort and depression/anxiety) to describe a range of health states. To convert the raw scores of the EQ-5D-3L into quality-adjusted life-years (QALYs), the Slovenian value set was used (elicited from the Slovenian population and based on preference-based valuation techniques). This value set was used as a proxy as none of the participating countries had value sets available. At each assessment, the QALY captures the participant’s quality of life with a score in which ‘1’ is considered perfect health and ‘0’ equals death, with negative values indicating health states worse than death.

### Analysis

With a minimum of n=90 per study arm, each of the five RECOVER-E trials was powered to detect a mean standardised difference of d≥0.33 as statistically significant at α≤0.05 (two-tailed) with a power of (1−β)=0.80 when WHODAS is evaluated in a baseline adjusted analysis of variance or a similarly specified linear mixed model. For details, we refer to our protocol papers.^8 20^

Intention-to-treat analysis was carried out with mixed models on the pooled dataset from all five trials. Assessments were ‘nested’ within participants and the participant ID number was used as level in the random part of the equation. The fixed part was modelled with outcome as a function of treatment condition, time and their interaction, adjusted for WHODAS at baseline. This analytical approach allows for missing values, so these were not imputed. To test our hypotheses, the condition×time interaction at the last follow-up (t_18_) was chosen as the parameter of interest. Since there were only five sites, this variable was not modelled as a level in the multilevel mixed model, but as a set of indicator variables in the fixed part of the equation to account for clustering of participants within sites.

Following the estimation of the mixed models, predictive marginal means were computed and graphed in a margin plot to visualise the impact of treatment on outcome over time. The analyses were repeated for all outcomes. All analyses were conducted in Stata V.17.0.^21^

### Sensitivity analysis

Two sensitivity analyses were performed, the first dealt with selection bias, which may have been introduced when dropout from the study was not random. Mixed models give unbiased estimates when due to study dropout information is missing completely at random, or when predictors of dropout are included as covariates in the model in which case one must rely on the missing at random (MAR) assumption.^22^ Since the estimates remained nearly identical when including predictors of dropout (a difference of less than 0.01 on a scale of 0–100), we decided to only report the outcomes of the more parsimonious models that did not include predictors of dropout. The second sensitivity analysis is related to COVID-19 incidence peaks, which impacted healthcare delivery in both the CMH and TAU conditions during data collection. Based on WHO data (https://covid19.who.int), we estimated that healthcare delivery was compromised during four periods: March–April 2020, July–August 2020, November 2020–April 2021 and August 2021–February 2022. Since these COVID-19 peaks were associated with reduced volumes of and changes in healthcare delivery modes,^23^ these peaks were also likely to have compromised the effectiveness of both the TAU and CMH interventions. We, therefore, added COVID-19 peak indicators in all assessments. These indicators were set at 1 when a participant happened to be assessed during one of these COVID-19 peaks or in the month following such a peak. These indicators were added as a time-varying confounder in the fixed part of the mixed models.^24^ The adjusted analyses were conducted for WHODAS Disability, WHODAS Response and Quality of Life.

## Findings

### Participants’ flow through the trial

**Figure 1:**
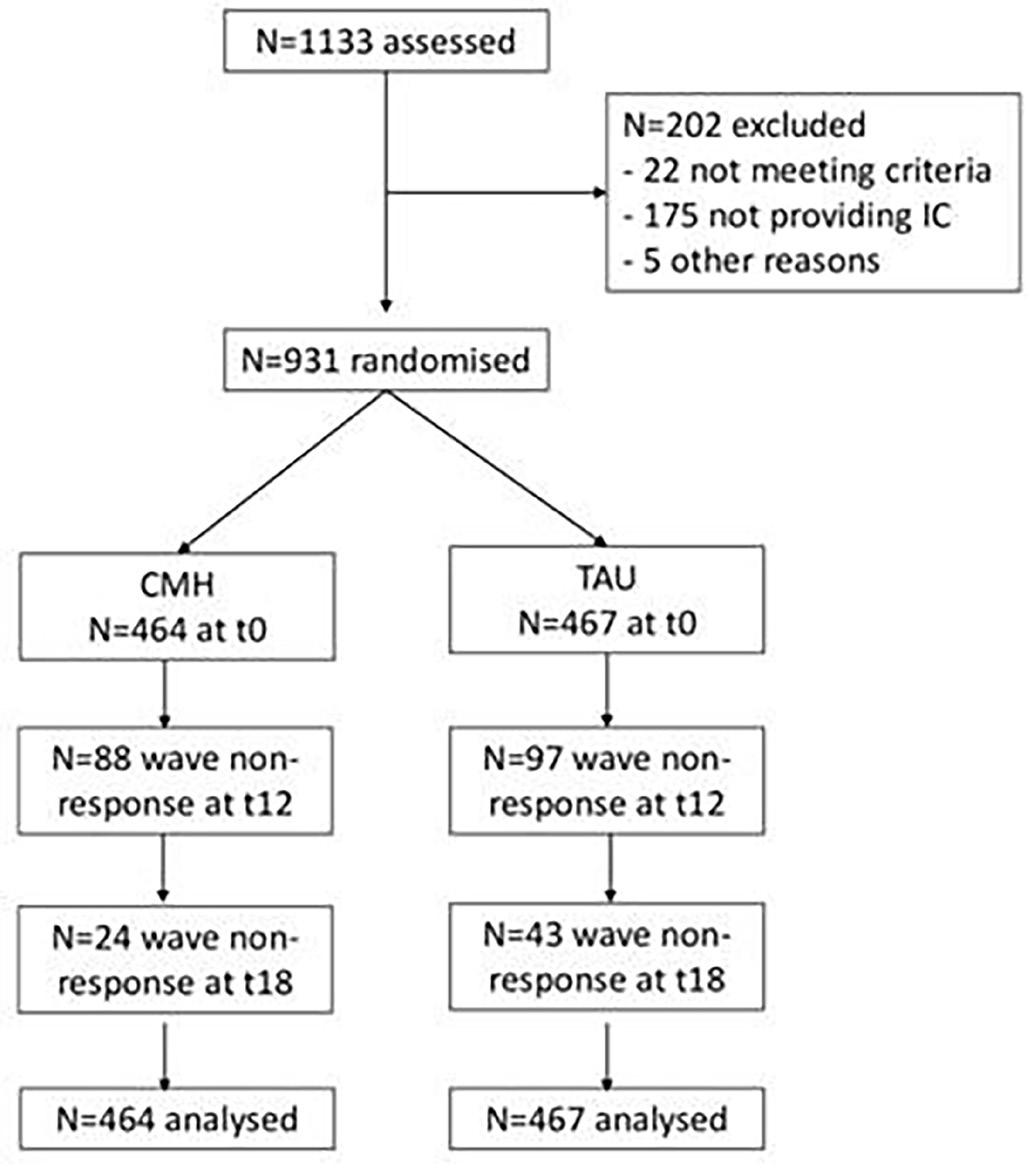
Participant flow. CMH, community mental healthcare; TAU, treatment as usual; IC, informed consent.

Recruitment of participants occurred between December 2018 and May 2020 (see online supplemental material 4). A total of 1133 service users were screened for eligibility; of these, 202 were excluded. 931 provided baseline data and were randomised to TAU (n=467) or CMH (n=464). Intention-to-treat analysis was based on the total sample size of N=931. Figure 1 depicts the participant flow, including study dropout.

At t_1_, 17 participants (3.6%) were assessed during a COVID-19 peak in TAU and N=14 (2.0%) in CMH. At t_12_, this changed into N=274 (65.6%) in TAU and N=275 (65.2%) in CMH, which became N=256 (64.8%) in TAU and N=260 (63.9%) in CMH at t_18_.

Between t_12_ and t_18_, four participants died (three in TAU and one in CMH, all judged not to be associated with the treatment they received). These people were included in the intention-to-treat analyses with their WHODAS score set at 100 (‘fully disabled’) and their EQ-5D quality of life score set at 0 (‘death’) at t_18_.

### Sample characteristics at baseline

The mean age of participants was 47.5 years (range 19–89), with half (52%) of them being female and 9.1% living with a partner. Most participants were unemployed, and a majority (70.8%) had a treatment history longer than 5 years, with schizophrenia being the most prevalent condition (46.8%). Baseline demographics were similar between both conditions except for gender, with a higher proportion of women in the TAU condition.

The mean WHODAS disability score was 35.1 (SD: 18.9) and mean EQ-5D-3L utility value was 0.66 (SD: 0.235) with no marked differences between the study arms. Looking at the distinct WHODAS domains, no baseline differences were found between arms except for the domains ‘getting along’ and ‘life activities’ for which the TAU condition scored somewhat higher compared with the CMH condition. An overview of baseline characteristics by group (CMH and TAU) and for the whole sample is provided as online supplemental material 5 (table S5.1), together with a separate overview by country (online supplemental tables S5.2–S5.6).

### Main outcome

**Figure 2:**
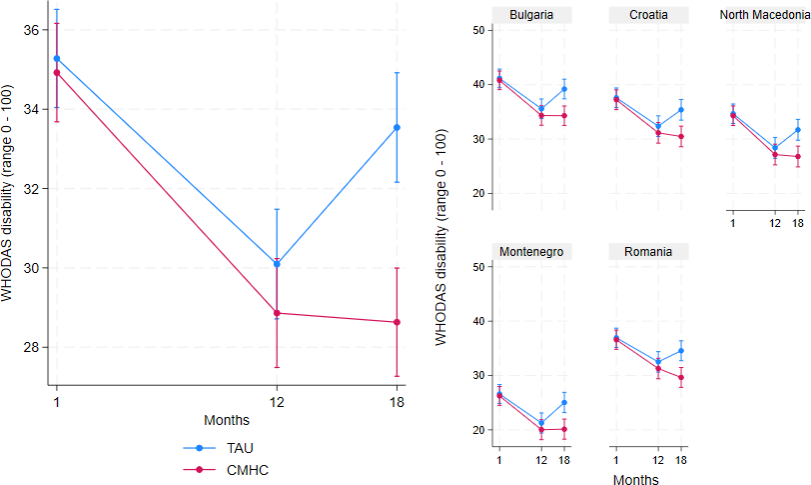
Changes in WHODAS disability by treatment condition over time and by country. CMHC, community mental health centre; TAU, treatment as usual; WHODAS, WHO Disability Assessment Schedule.

Figure 2 depicts the means of WHODAS disability by condition over time as estimated under the mixed model adjusted for baseline differences of WHODAS disability.

Both CMH and TAU started with similar WHODAS mean scores at t_1_ and then followed a similar downward trajectory to t_12_, then diverged with consistently less disability in CMH participants, while disability levels veered back to worse scores in TAU, leading to a significant between-group treatment effect at t_18_ (b=−4.55, SE=1.21, z=−3.75, p<0.001), favouring CMH over TAU. Each of the participating countries followed the same pattern, which attests to the robustness of the main finding and might be indicative of the transferability of CMH across local health system ecologies and contexts.

### Secondary outcomes

**Figure 3:**
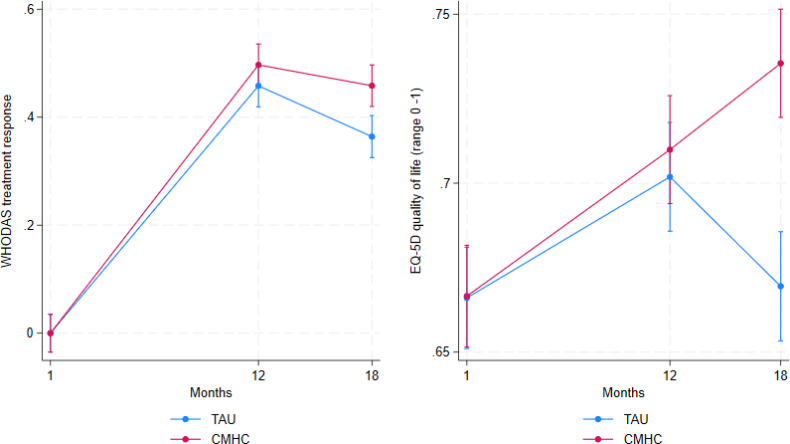
WHODAS treatment response and EQ-5D-3L utility by condition over time. CMHC, community mental health centre; EQ-5D-3L, EuroQoL with 5 Dimensions-3 Levels; TAU, treatment as usual; WHODAS, WHO Disability Assessment Schedule.

As indicated in figure 3, both conditions had a similar treatment response rate at t_12_, with CMH sustaining it towards t_18_ while treatment response rates dropped in TAU, resulting in a 6.4% difference between CMH and TAU at t_18_. In the base-case analysis, this difference reached almost significance (b=0.06, SE=0.03, z=1.94, p=0.052) in the main analysis but became statistically significant in the sensitivity analyses where adjustments were made for the impact of COVID-19 peaks (see below).

Improvements in health-related quality of life by study arm over time are depicted in the right-hand panel of figure 3, where both CMH and TAU started off with a similar mean utility of 0.67, then improved quality of life at the same rate to a mean utility of about 0.70 at t_12_. After t_12_, TAU dropped back to almost baseline level (0.67), while CMH continued to improve to 0.74, resulting in a statistically significant between-group treatment effect of 0.07 utility (b=0.07, SE=0.014, z=4.56, p<0.001) indicating better health-related QoL in CMH as compared with TAU at t_18_.

Table 1 presents the treatment effects on each of the distinct WHODAS domains, showing that CMH was more successful than TAU in reducing disability in all WHODAS domains, except in the domain of Activities (b= –3.14, p=0.058).

**Table 1:** Effect of CMH versus TAU on WHODAS domains at last follow-up (t_18_)

WHODAS domains	b	SE	Z	P value	95 % CI lower	95% CI upper
Cognition	−4.84	1.57	−3.09	0.002	−7.90	−1.77
Mobility	−5.09	1.58	−3.21	0.001	−8.19	−1.98
Self-care	−3.29	1.52	−2.16	0.031	−6.27	−0.30
Getting along	−3.53	1.68	−2.10	0.036	−6.83	−0.24
Activities	−3.14	1.65	−1.90	0.058	−6.38	0.10
Participation	−5.21	1.57	−3.32	0.001	−8.29	−2.13
WHODAS total	−4.55	1.21	−3.75	<0.001	−6.93	−2.17

CMH, community mental healthcare; TAU, treatment as usual; WHODAS, WHO Disability Assessment Schedule.

The results presented in figures2  3 are provided as statistics in online supplemental material 6.

### Sensitivity analyses

The effect estimates (b-coefficients) from both analyses were similar or slightly better for CMH in the sensitivity analysis on the impact of COVID-19. These differences are minute and do not substantially change interpretation of our findings. However, the p value for response came down from p=0.052 in the base-case analysis to p=0.012 in the sensitivity analysis (see online supplemental material 7).

## Discussion

### Principal findings

Pooled data (N=931) from five RCTs were used to evaluate the effectiveness of recovery-oriented CMH compared with institution-based care across five sites in SEE. We found that CMH had significantly lower WHODAS disability scores (b=–4.55, p≤0.001) at 18-month follow-up compared with institution-based TAU. This finding was consistent across all five sites and with all six WHODAS outcome domains of personal and social functioning. In addition, we found that CMH had a 6.4% better treatment response rate than TAU (p=0.052 in the main analysis and p=0.012 in the COVID adjusted analysis), which is noteworthy because 70% of the service users in our study had a treatment history longer than 5 years for schizophrenia, bipolar disorder and severe depression. Finally, the differential treatment effect on EQ-5D health-related quality of life was 0.07 (p<0.001), which translates into an additional health gain of 25 days in full health in those receiving CMH. All findings were consistent with sensitivity analyses in which we adjusted for the impact of COVID-19.

In short, our study provides evidence of the benefits of CMH delivered by CMH teams with the mandate and practical means to provide recovery-oriented services in improving functioning, reducing disability and improving quality of life compared with traditional, institution-based care. Such evidence is needed in the region of SEE to continue driving reforms focused on deinstitutionalisation and aimed at personal and social recovery and improving health-related quality of life in those most in need of it.

### Findings in context

In 2017, a meta-analysis was published on the effectiveness of CMH in low-income and middle-income countries.^25^ This meta-analysis was based on 1580 people with schizophrenia participating in 11 RCTs across 5 countries (China, India, Iran, South Africa and Turkey). A subset of seven RCTs specifically compared community-based psychosocial intervention (N=496) with TAU (N=367) and found a strong effect favouring CMH on symptom severity (standard mean difference, SMD: 0.95, 95% CI: 0.28 to 1.61) and improved functioning (SMD: 1.12, 95% CI: 0.25 to 2.00) at 18 months postintervention.

Since then, three more recent RCTs appeared on the effectiveness of CMH in low-income and middle-income countries.^26^,^28^ One of these is about people with schizophrenia in rural areas.^28 28^ This study showed that community-based healthcare (N=73) contributes to better outcomes than institutional care (N=73) delivering better WHODAS functioning (SMD: 0.35, 95% CI: 0.02 to 0.67) and lesser severity of psychotic symptoms (SMD: 0.36, 95% CI: −0.01 to 0.70) at 12 months postbaseline. Two other trials were conducted in mainland China,^26 27^ both reporting significantly better health outcomes under CMH than TAU.

Overall, the data from low-income and middle-income countries suggest significant and sustained effectiveness of CMH with varying content (ie,psychoeducation, rehabilitation, case-management, assertive community treatment) for schizophrenia and other SMI. This is consistent with our own findings.

While notable transformations of mental health systems with implementation of community-based models of care took place in the last years in other low-middle-income countries in Europe, like Moldova and Czechia,^29 30^ research documenting their outcomes for people with SMI is not yet available. Therefore, our findings bring in important evidence previously not available for low-income and middle-income settings in SEE, that CMH teams can be implemented and deliver beneficial health outcomes for people with SMI.

### Strength and limitations

The study has the following strengths. First, a pragmatic approach was chosen to implement and evaluate CMH in the five sites. While this may reduce internal validity (eg, due to a lack of masking), the corresponding results may have greater ecological validity to enact healthcare system change under real-life conditions. Second, each of the five trials was sufficiently powered to detect clinically relevant and statistically significant change in the participants. This provided each of the participating countries with a sound evidence base for scaling up CMH.

We also acknowledge several limitations of this study. The first relates to study dropout. In our sensitivity analysis, effect estimates adjusted for observed predictors of dropout differed from the unadjusted estimates only at the fifth decimal place, suggesting that any bias due to observed factors was negligible. Nonetheless, unobserved correlates of dropout could still have biased the estimates.

Second, the COVID-19 peaks that occurred during the trial period affected treatment delivery and outcomes. However, sensitivity analyses adjusted for these disruptions closely replicated the main findings, supporting the robustness of our results. Third, the outcomes were based on self-report measures. Although self-report may introduce bias, subjective experiences such as disability and quality of life are inherently introspective and cannot be reliably assessed through other means.

Finally, treatment fidelity was not monitored or evaluated. Consequently, the CMH intervention may not have been implemented exactly as intended across all five sites. Therefore, we can draw conclusions about the effectiveness of CMH under naturalistic conditions, but not about its efficacy under controlled conditions, the intended scope of these pragmatic trials.

### Conclusions

One of the major challenges in reforming mental health systems in SEE has been the integration, sustainability and scaling up of comprehensive and evidence-based practices into CMH services.^2^ In the five RECOVER-E sites, a situation analysis identified that although previous national initiatives to strengthen mental health care had generated broad support for referral to CMH services, this support has not been matched by the actual availability of such services. This study provides empirical evidence on the extent to which CMH services delivered by multidisciplinary community mental health teams

influence clinical outcomes when implemented in five different contexts that share similar historical and mental health system characteristics. Our results show that CMH services can yield positive clinical outcomes at various stages of mental health system transformation, whether in settings with no prior CMH infrastructure or in those where pilot initiatives have already been introduced. Furthermore, beneficial clinical effects of receiving recovery-oriented care in one’s community were observed despite variation in human and financial resources for mental health across sites, underscoring the adaptability of the model to local circumstances and contexts.

### Implications

The results of this study hold both scientific and public health relevance. We demonstrated that mental health services transformation towards CMH is feasible and can yield tangible, sustained benefits over an 18-month follow-up period. Moreover, our study gives actionable input to decision-makers in the five participating sites. They can be encouraged to scale up evidence-based CMH models with the dual ambition of improving the lives of service users and creatively leveraging the resources of local health ecosystems, while remaining determined to overcome long-standing system level challenges.

## Supplementary material

10.1136/bmjgh-2024-018594 online supplemental file 1

10.1136/bmjgh-2024-018594 online supplemental file 2

10.1136/bmjgh-2024-018594 online supplemental file 3

10.1136/bmjgh-2024-018594 online supplemental file 4

10.1136/bmjgh-2024-018594 online supplemental file 5

10.1136/bmjgh-2024-018594 online supplemental file 6

10.1136/bmjgh-2024-018594 online supplemental file 7

## Data Availability

Data are available on reasonable request. All data relevant to the study are included in the article or uploaded as supplementary information.
