# Supplementary material for: Community versus institutionalised care for people with severe mental illness in five countries in Southeast Europe: pooled analysis of five randomised trials
Source: BMJ Glob Health. 2025 Oct 23;10(10):e018594. doi: 10.1136/bmjgh-2024-018594 (PMC12551481; doi:10.1136/bmjgh-2024-018594)
Supplement: online supplemental file 2 [file bmjgh-10-10-s002.docx]

**
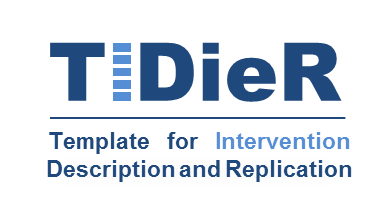
The TIDieR (Template for Intervention Description and Replication) Checklist*:**

Information to include when describing an intervention and the location of the information

| **Item number** | **Item** |  |
| --- | --- | --- |
|  |  | Primary paper  (intervention description, page 5) |
|  | **BRIEF NAME** |  |
| **1.** | Provide the name or a phrase that describes the intervention. | RECOVER-E: large-scale implementation of community based mental health care for people with severe and Enduring mental ill health in europe |
|  | **WHY** |  |
| **2.** | Describe any rationale, theory, or goal of the elements essential to the intervention. | Community-based mental services that integrate psychological, social and pharmacological support in and around a person’s context and based on their needs have shown improved health and social outcomes. Much of the research on community mental health service delivery models has been based in high-income countries. Community-based mental health services have been piloted in numerous countries in Central and Eastern Europe as part of the process of deinstitutionalization; however, barriers like limited resources and a lack of evidence-based care pathways hinder large-scale implementation. Furthermore, care in Central and Eastern Europe is often monodisciplinary, and often does not take the full context and needs of the person with severe mental illness. Care is often focused on treating symptoms and on deficits and limitations, rather than focusing on recovery and on strengths. This intervention therefore focused on the implementation of multidisciplinary community mental health teams delivering community-based services that apply recovery-oriented care principles in five sites in five countries, supporting the transition from hospital to community-based care. |
|  | **WHAT** |  |
| **3.** | Materials: Describe any physical or informational materials used in the intervention, including those provided to participants or used in intervention delivery or in training of intervention providers. Provide information on where the materials can be accessed (e.g. online appendix, URL). | The intervention group (community mental health team, or CMHT) received a training programme on community mental health and in working as part of a multidisciplinary community mental health team, provided in English and the local language. More information can be found [here](https://ijmhs.biomedcentral.com/articles/10.1186/s13033-020-00361-y) & [here](https://ec.europa.eu/research/participants/documents/downloadPublic?documentIds=080166e5ba54a426&appId=PPGMS).  [Documents download module](https://ec.europa.eu/research/participants/documents/downloadPublic?documentIds=080166e5ba54a426&appId=PPGMS) (European Commission webpage, link to RECOVER-E project)  [Towards community-based and recovery-oriented care for severe mental disorders in Southern and Eastern Europe: aims and design of a multi-country implementation and evaluation study (RECOVER-E) \| International Journal of Mental Health Systems \| Full Text](https://ijmhs.biomedcentral.com/articles/10.1186/s13033-020-00361-y): Specifically, the Intervention sub-heading. |
| **4.** | Procedures: Describe each of the procedures, activities, and/or processes used in the intervention, including any enabling or support activities. | Several implementation strategies were applied to support implementation of the CMHT. These included: 1) training (one week in-country and one week in the Netherlands, and refresher trainings based on need); 2) peer support: specific training for people with lived experience to become paid members of the CMHT as peer workers, and ongoing mentoring and coaching for peer workers; 3) mentoring and supervision through monthly implementation calls; 4) CMHT roadmaps. The five CMHT (one per site) each created a roadmap before implementation, which detailed the goals and milestones of the CMHT, roles and responsibilities of the team members, and what is needed to achieve CMHT goals, in terms of training, capacity building, or infrastructure (e.g. a laptop and projector for the CMHT to review shared caseload in routine team meetings, or access to public transportation or a car from the mental health service to do home visits), 5) feedback on applying recovery-oriented and strength-based care principles and approaches into work; and 6) support in developing a plan for sustaining the CMHT after the project ends, focusing on sustainability. |
|  | **WHO PROVIDED** |  |
| **5.** | For each category of intervention provider (e.g. psychologist, nursing assistant), describe their expertise, background and any specific training given. | Intervention providers include a multidisciplinary team of professionals, including the core professional groups: social workers, nurses, psychologists, peer workers (persons with lived experience of a severe mental illness), and psychiatrists. Some countries with more human resources for mental health may have added additional professionals such as job coaches. CMHTs received two full weeks of training, one week in the Netherlands (to understand how the CMHT model works in practice by shadowing peer-professionals and visiting mental health services), and one week in each of the site countries (Montenegro, North Macedonia, Bulgaria, Croatia and Romania). Training was provided by psychiatrists, psychologists, peer workers, social workers and psychiatric nurses with expertise in delivering training in community mental health and how to work in a CMHT. These two weeks of training were done face-to-face prior to implementation of the intervention in each of the 5 sites. Follow-up training sessions on specific clinical skills, team-aspects of the CMHTs, were provided by the same trainers mentioned above, online, during the COVID-19 pandemic. Topics for follow-up trainings was assessed during routine needs assessment documents issued to the professionals in the CMHTs in the 5 countries and also discussed in monthly Implementation team meetings (for all implementers from the five sites, plus trainers and the coordinating organisation of the project). |
|  | **HOW** |  |
| **6.** | Describe the modes of delivery (e.g. face-to-face or by some other mechanism, such as internet or telephone) of the intervention and whether it was provided individually or in a group. | Delivery of the services provided by the CMHTs was provided in an individual format to service users with severe mental illness, and were provided face-to-face or via telephone call. As the intervention also partially took place during the COVID-19 pandemic, some services were done through telemedicine via phone. |
|  | **WHERE** |  |
| **7.** | Describe the type(s) of location(s) where the intervention occurred, including any necessary infrastructure or relevant features. | The intervention occurred in clinical settings (e.g. meeting rooms or consultation rooms in outpatient departments of general hospitals or psychiatric hospitals, in service user’s homes (home visits), online/by phone (during the COVID-19 pandemic) and in some cases in other public locations such as public parks, in agreement by the service user and the CMHT professional. |
|  | **WHEN and HOW MUCH** |  |
| **8.** | Describe the number of times the intervention was delivered and over what period of time including the number of sessions, their schedule, and their duration, intensity or dose. | The intervention was a routine clinical service offered, on an as needed basis by service users. The nature of the intervention is that the CMHT works flexibly according to service user needs and can upscale or downscale intensity of contact and follow-up with service users. This is consistent with the Flexible Assertive Community Treatment Approach (FACT), of which the principles were adapted for use in the 5 CMHTs in the 5 countries in the RECOVER-E project. |
|  | **TAILORING** |  |
| **9.** | If the intervention was planned to be personalised, titrated or adapted, then describe what, why, when, and how. | The intervention was tailored in several ways. First, it was tailored to the country context and the resource-levels of the specific mental health services and systems in the 5 sites in the 5 countries. This tailoring was done after an extensive formative evaluation period when a situation analysis and needs assessment were carried out in the 5 sites, which led to a roadmap for training needs for CMHT professionals and specificities of each site related to the CMHT. Second, the intervention is a flexible form of a community mental health team, meaning that care and support provided by the team was tailored to the needs and recovery goals of service users in each of the five sites with severe mental illness (schizophrenia, bipolar disorder or severe depression). Third, a standard set of implementation strategies were described in the protocol paper, which included training, refresher training sessions and supervision sessions, and CMHT roadmaps (plans, made by each CMHT in each site, detailing what the goal of the CMHT was in, available skillsets and expertise, roles and responsibilities of the CMHT within the mental health system context, and needs for support or training from CMHT professionals). Some of the implementation strategies were needed more in some sites than in others, so tailoring here meant that some sites made more use of refresher sessions on specific clinical skills to aid in implementation of the CMHT service, compared to other sites. These were all documented in implementation logs maintained by the project team, discussed in monthly implementation calls. |
|  | **MODIFICATIONS** |  |
| **10.^ǂ^** | If the intervention was modified during the course of the study, describe the changes (what, why, when, and how). | As the intervention was partially delivered in the 5 sites during the COVID-19 pandemic (March 2020- until the end of the implementation of the intervention, 31.12.2021), unanticipated modifications to the intervention included shifting from home visits and face-to-face care to telephone consultations with CMHT professionals or visits/consultations employing physical distancing and PPE equipment, depending on local lockdown measures. |
|  | **HOW WELL** |  |
| **11.** | Planned: If intervention adherence or fidelity was assessed, describe how and by whom, and if any strategies were used to maintain or improve fidelity, describe them. | Only fidelity to the CMHT’s orientation towards recovery-oriented care and strength-based approaches in mental health were assessed, at baseline and at 12 month follow-up, by independent researchers in each of the 5 sites who were not involved in implementation of the intervention, guided by independent researchers based at the University of Heidelberg, who led the project’s work package on Research Evaluation. |
| **12.^ǂ^** | Actual: If intervention adherence or fidelity was assessed, describe the extent to which the intervention was delivered as planned. | With respect to the CMHT’s orientation towards recovery-oriented care and strength-based approaches, the intervention contributed to improvements in provision of recovery-oriented care, adoption of recovery-oriented care principles in practice, and in employing strength-based rather than deficit or disability based approaches. |

** **Authors** - use N/A if an item is not applicable for the intervention being described. **Reviewers** – use ‘?’ if information about the element is not reported/not sufficiently reported.

† If the information is not provided in the primary paper, give details of where this information is available. This may include locations such as a published protocol or other published papers (provide citation details) or a website (provide the URL).

ǂ If completing the TIDieR checklist for a protocol, these items are not relevant to the protocol and cannot be described until the study is complete.

* We strongly recommend using this checklist in conjunction with the TIDieR guide (see *BMJ* 2014;348:g1687) which contains an explanation and elaboration for each item.

* The focus of TIDieR is on reporting details of the intervention elements (and where relevant, comparison elements) of a study. Other elements and methodological features of studies are covered by other reporting statements and checklists and have not been duplicated as part of the TIDieR checklist. When a **randomised trial** is being reported, the TIDieR checklist should be used in conjunction with the CONSORT statement (see [www.consort-statement.org](http://www.consort-statement.org)) as an extension of **Item 5 of the CONSORT 2010 Statement.** When a **clinical trial** **protocol** is being reported, the TIDieR checklist should be used in conjunction with the SPIRIT statement as an extension of **Item 11 of the SPIRIT 2013 Statement** (see [www.spirit-statement.org](http://www.spirit-statement.org)). For alternate study designs, TIDieR can be used in conjunction with the appropriate checklist for that study design (see [www.equator-network.org](http://www.equator-network.org)).
