# Supplementary material for: Community versus institutionalised care for people with severe mental illness in five countries in Southeast Europe: pooled analysis of five randomised trials
Source: BMJ Glob Health. 2025 Oct 23;10(10):e018594. doi: 10.1136/bmjgh-2024-018594 (PMC12551481; doi:10.1136/bmjgh-2024-018594)
Supplement: online supplemental file 3 [file bmjgh-10-10-s003.docx]

**Reflexivity Statement**

Reflexivity involves a set of practices through which researchers critically examine how their subjectivity and context influence the research process (Olmos-Vega, Stalmeijer, Varpio, & Kahlke, 2022). In this statement, we outline how we engaged in reflexivity, following the structure provided by Jamieson, Govaart, & Pownall (2023).

**Study Conceptualization**

**1. Addressing Local Research and Policy Priorities**

This study emerged from an ongoing collaboration on community mental health services across multiple countries. Two co-authors had prior experience working with partners in Croatia and Montenegro on similar mental health projects, while one author had an established collaboration with mental health policymakers across all five participating countries. Before data collection, a comprehensive needs assessment was conducted in Romania, Bulgaria, North Macedonia, Croatia, and Montenegro to ensure the project aligned with local needs and resource levels. Additionally, all participating countries demonstrated policy readiness to implement the project as part of their mental health system strengthening efforts.

**2. Involvement of Local Researchers in Study Design**

Each country had designated clinical and research leads responsible for designing the local trial. These research leads could seek methodological guidance from Work Package leaders based in Germany and the Netherlands when necessary. Local researchers also played a key role in the formative phase, contributing to recruitment strategy development, selection of trial measures, and the decision to implement a staggered trial approach across the five countries.

**3. Funding Support for Local Research Teams**

Funding from the larger project supported various aspects of the study, including the needs assessment, research activities (recruitment, data management, analysis, and dissemination), and capacity-building initiatives. Multiple workshops on research methodology were provided, both in-person and virtually, to support researchers across the five sites, with additional guidance available upon request.

**Data Acquisition and Analysis**

**4. Acknowledgment of Research Staff Involved in Data Collection**

All country research leads and contributors are included in the RECOVER-E group authorship list, with additional researchers acknowledged in the manuscript.

**5. Data Access for Research Partners**

Each country team retained ownership of its data. This manuscript presents aggregated results from the five trials. Researchers from high-income institutions had access to anonymized datasets to assist with data cleaning and analysis. Cleaned datasets and analysis codes were shared with each country team for independent use.

**6. Capacity Building in Data Analysis**

Each country team conducted its own data analysis using standardized codes and interpreted the results presented in this manuscript. Researchers from high-income institutions provided ongoing methodological training during and after the project.

**Data Interpretation**

**7. Collaboration in Data Interpretation**

High-income country researchers collaborated with each of the five country teams to analyze and interpret data. Findings were presented, discussed, and contextualized during the final project meeting.

**8. Support for Developing Writing Skills**

Each country team prepared a summary of its findings and contributed to manuscript drafts. All authors, representing the five countries, reviewed and approved the final version of the manuscript.

**9. Dissemination of Findings to Address Local Needs**

Each country team determined how to disseminate its trial findings. Some shared results with Ministries of Health to support policymaking, while others engaged professional associations (e.g., national psychiatric associations) to highlight the impact of multidisciplinary community mental health teams, including peer workers.

**Authorship and Capacity Building**

**10. Recognition of LMIC Leadership and Contribution**

All country research leads and Work Package leaders involved in the research are included in the RECOVER-E group authorship list.

**11. Inclusion of Early-Career Researchers in Authorship**

Country research leads included early-career, mid-career, and senior researchers, all of whom are represented in the authorship list. The main authorship also reflects a mix of experience levels, including PhD candidates, recent graduates, and senior faculty members.

**12. Gender Balance in Authorship**

The authorship list reflects gender balance. While gender ratios varied across country teams, overall representation was equitable.

**13. Training and Capacity Building for LMIC Researchers**

This study is part of the RECOVER-E project, which involved diverse organizations spanning clinical, research, policy, and lived experience expertise. LMIC co-authors were country leads, clinician-researchers, or other contributors involved in study design, analysis, interpretation, and dissemination. Training opportunities included a one-week in-person research program, virtual mentorship, and topic-specific webinars and workshops tailored to country needs.

**14. Contributions to Local Research Infrastructure**

Project funding supported both clinical implementation (e.g., laptops, projectors for case review meetings) and research infrastructure (e.g., translation of tools, software acquisition).

**Additional Reflexivity Reflections**

**Research Questions**

The research questions were informed by prior experiences of the co-authors and project partners. Previous projects, particularly in Croatia and Montenegro, helped shape the study and its expansion to other countries. The research team included individuals both "within" (researchers from implementing sites) and "outside" (persons with severe mental illness) the population of interest. A peer expert, who co-authored this paper, played a crucial role in developing training for mental healthcare providers, ensuring the integration of lived experience. While the trainer’s cultural background may have introduced bias, their lived experience was a vital component of the project.

**Data Collection**

Data collection was integrated into the routine activities of mental healthcare staff. Beyond this publication, further efforts are needed to make findings accessible to participants. One co-author (GM), who leads a service user organization, intends to use these results to engage national and international advocacy groups, indirectly benefiting those who contributed their data.

**Conclusions and Framing**

Our conclusions may reflect the research team’s perspective on what is "needed" in mental healthcare in these five countries. The policy focus of the study stems from its goal of informing governmental decisions on deinstitutionalization and community-based care.

**References**

- Olmos-Vega, F. M., Stalmeijer, R. E., Varpio, L., & Kahlke, R. (2022). A practical guide to reflexivity in qualitative research: AMEE Guide No. 149. *Medical Teacher, 45*(3), 241–251. https://doi.org/10.1080/0142159X.2022.2057287
- Jamieson, M. K., Govaart, G. H., & Pownall, M. (2023). Reflexivity in quantitative research: A rationale and beginner's guide. *Social and Personality Psychology Compass, 17*(4), e12735. https://doi.org/10.1111/spc3.12735
