# Supplementary material for: Community versus institutionalised care for people with severe mental illness in five countries in Southeast Europe: pooled analysis of five randomised trials
Source: BMJ Glob Health. 2025 Oct 23;10(10):e018594. doi: 10.1136/bmjgh-2024-018594 (PMC12551481; doi:10.1136/bmjgh-2024-018594)
Supplement: online supplemental file 4 [file bmjgh-10-10-s004.pdf]

Supplemental Material 4

Table S4.1 Data collection phases by country

Project site	Start of study	T1 first patient (12-months follow up)	T1 last patient	T2 first patient (18-months follow up)	T2 last patient	Data collection completed
Croatia	December 2018	December 2019	April 2021	June 2020	October 2021	August 2021
Montenegro	February 2019	February 2020	December 2020	August 2020	June 2021	June 2021
North Macedonia	June 2019	June 2020	February 2021	December 2020	August 2021	August 2021
Romania	April 2019	April 2020	April 2021	October 2020	October 2021	October 2021
Bulgaria	October 2019	October 2020	April 2021	April 2021	October 2021	October 2021
