# Supplementary material for: Community versus institutionalised care for people with severe mental illness in five countries in Southeast Europe: pooled analysis of five randomised trials
Source: BMJ Glob Health. 2025 Oct 23;10(10):e018594. doi: 10.1136/bmjgh-2024-018594 (PMC12551481; doi:10.1136/bmjgh-2024-018594)
Supplement: online supplemental file 5 [file bmjgh-10-10-s005.pdf]

Supplemental Material 5

Table S5.1. *Baseline characteristics of the sample by CMH and TAU condition*

	**Community mental care (N= 464)**	**Treatment as usual (N= 467)**	**Both conditions (N= 931)**
Demographics			
. Age, mean (Sd)	47.3 (12.3)	47.8 (12.9)	47.5 (12.6)
. Female gender, N (%)	253 (54.5%)	230 (49.3%)	483 (51.9%)
. Has a partner, N (%)	44 (9.5%)	41 (8.8%)	85 (9.1%)
. Employed, N (%)	106 (22.8%)	95 (20.3%)	201 (21.6%)
. Above average income, N (%)	23 (5.0%)	13 (2.8%)	36 (3.9%)
Education			
. Primary, N (%)	98 (21.2%)	108 (23.2%)	206 (22.2%)
. Secondary, N (%)	208 (44.9%)	199 (42.8%)	407 (43.9)
. Lower vocational, N (%)	49 (10.6%)	65 (14.0%)	114 (12.3%)
. Higher vocational and academic, N (%)	108 (23.3%)	93 (20.0%)	201 (21.7%)
ICD-10 diagnosis and history			
. Schizophrenia, N (%)	224 (48.3%)	212 (45.4%)	436 (46.8%)
. Bipolar, N (%)	53 (11.4%)	59 (12.6%)	112 (12.0%)
. Major depression, N (%)	99 (21.3%)	92 (19.7%)	191 (20.5%)
. Treatment history > 5 years, N (%)	328 (70.7%)	331 (70.9%)	659 (70.8%)
WHODAS functional disability			
. Disability, Mean (SD)	34.4 (19.4)	35.8 (18.4)	35.1 (18.9)
. Cognition, Mean (SD)	34.0 (26.8)	34.4 (24.2)	34.2 (25.5)
. Mobility, Mean (SD)	23.8 (26.1)	25.2 (25.8)	24.5 (25.9)
. Self-care, Mean (SD)	17.7 (22.9)	19.5 (23.7)	18.6 (23.3)
. Getting along, Mean (SD)	37.0 (26.5)	40.2 (25.6)	38.6 (26.1)
. Life activities, Mean (SD)	34.9 (26.9)	38.2 (26.9)	36.6 (27.0)
. Participation, Mean (SD)	48.7 (23.0)	48.5 (20.7)	48.6 (21.9)
EQ-5D-3L quality of life			
. Quality of Life, Mean (SD)	0.668 (0.236)	0.664 (0.234)	0.666 (0.235)

Table S5.2 Summary Table for Bulgaria

	Treatment as usual (N= 99)	Community mental care (N= 101)	Overall (N= 200)
**Age in years at baseline**			
Mean (SD)	45.5 (12.1)	44.5 (11.2)	45.0 (11.6)
**Female gender**			
Mean (SD)	0.485 (0.502)	0.525 (0.502)	0.505 (0.501)
**Living with/out a partner**			
Mean (SD)	0.0707 (0.258)	0.0990 (0.300)	0.0850 (0.280)
**Employment status**			
Mean (SD)	0.101 (0.303)	0.178 (0.385)	0.140 (0.348)
**Above average income**			
Mean (SD)	0.0101 (0.101)	0.0594 (0.238)	0.0350 (0.184)
**Education**			
Primary education	39 (39.4%)	18 (17.8%)	57 (28.5%)
Secondary education	39 (39.4%)	33 (32.7%)	72 (36.0%)
Lower vocational education	5 (5.1%)	16 (15.8%)	21 (10.5%)
Higher vocational and academic	16 (16.2%)	34 (33.7%)	50 (25.0%)
**ICD-10 psychotic spectrum disorders**			
Mean (SD)	0.798 (0.404)	0.743 (0.439)	0.770 (0.422)
**ICD-10 bipolar disorder**			
Mean (SD)	0.121 (0.328)	0.218 (0.415)	0.170 (0.377)
**ICD-10 major depressive disorder**			
Mean (SD)	0.0808 (0.274)	0.0396 (0.196)	0.0600 (0.238)
**Long treatment history > 5 years**			
Mean (SD)	0.758 (0.431)	0.723 (0.450)	0.740 (0.440)
**WHODAS 2.0 at t1**			
Mean (SD)	37.9 (17.5)	44.7 (18.2)	41.4 (18.1)
**imputed Cognition – understanding & communicating**			
Mean (SD)	38.6 (21.3)	51.3 (24.5)	45.0 (23.8)
**imputed Mobility – moving & getting around**			
Mean (SD)	25.3 (22.8)	26.9 (27.7)	26.1 (25.3)
**imputed Self-care – hygiene, dressing, eating & staying alone**			
Mean (SD)	23.1 (24.7)	23.9 (27.1)	23.5 (25.9)
**imputed Getting along– interacting with other people**			
Mean (SD)	45.6 (21.8)	49.2 (22.5)	47.4 (22.2)
**imputed Life activities– domestic responsibilities, leisure, work & school**			
Mean (SD)	44.1 (25.1)	49.4 (26.3)	46.8 (25.8)

Table S5.2 Summary Table for Bulgaria

	Treatment as usual (N= 99)	Community mental care (N= 101)	Overall (N= 200)
**imputed Participation– joining in community activities**			
Mean (SD)	45.6 (19.0)	55.7 (18.5)	50.7 (19.4)
**EQ-5D-3L utility at t1**			
Mean (SD)	0.697 (0.229)	0.652 (0.233)	0.674 (0.232)

Table S5.3. Summary Table for Croatia

	Treatment as usual (N= 86)	Community mental care (N= 83)	Overall (N= 169)
**Age in years at baseline**			
Mean (SD)	43.0 (13.5)	41.4 (11.6)	42.2 (12.6)
**Female gender**			
Mean (SD)	0.512 (0.503)	0.530 (0.502)	0.521 (0.501)
**Living with/out a partner**			
Mean (SD)	0.0233 (0.152)	0.0482 (0.215)	0.0355 (0.186)
**Employment status**			
Mean (SD)	0.279 (0.451)	0.229 (0.423)	0.254 (0.437)
**Above average income**			
Mean (SD)	0.0116 (0.108)	0.0241 (0.154)	0.0178 (0.132)
**Education**			
Primary education	11 (12.8%)	8 (9.6%)	19 (11.2%)
Secondary education	37 (43.0%)	42 (50.6%)	79 (46.7%)
Lower vocational education	15 (17.4%)	6 (7.2%)	21 (12.4%)
Higher vocational and academic	23 (26.7%)	27 (32.5%)	50 (29.6%)
**ICD-10 psychotic spectrum disorders**			
Mean (SD)	0.663 (0.476)	0.639 (0.483)	0.651 (0.478)
**ICD-10 bipolar disorder**			
Mean (SD)	0.0814 (0.275)	0.0482 (0.215)	0.0651 (0.247)
**ICD-10 major depressive disorder**			
Mean (SD)	0.128 (0.336)	0.145 (0.354)	0.136 (0.344)
**Long treatment history > 5 years**			
Mean (SD)	0.767 (0.425)	0.747 (0.437)	0.757 (0.430)
**WHODAS 2.0 at t1**			
Mean (SD)	41.2 (20.8)	38.0 (20.5)	39.6 (20.7)
**imputed Cognition – understanding & communicating**			
Mean (SD)	44.6 (27.5)	38.0 (30.1)	41.4 (28.9)
**imputed Mobility – moving & getting around**			
Mean (SD)	26.7 (29.7)	22.2 (25.3)	24.5 (27.6)
**imputed Self-care – hygiene, dressing, eating & staying alone**			
Mean (SD)	17.0 (25.5)	16.3 (19.3)	16.7 (22.6)
**imputed Getting along– interacting with other people**			
Mean (SD)	45.2 (29.0)	43.1 (31.2)	44.2 (30.0)
**imputed Life activities– domestic responsibilities, leisure, work & school**			
Mean (SD)	44.0 (32.2)	39.9 (29.4)	42.0 (30.8)

Table S5.3. Summary Table for Croatia

	Treatment as usual (N= 86)	Community mental care (N= 83)	Overall (N= 169)
**imputed Participation– joining in community activities**			
Mean (SD)	54.0 (26.3)	53.6 (27.1)	53.8 (26.6)
**EQ-5D-3L utility at t1**			
Mean (SD)	0.579 (0.260)	0.639 (0.225)	0.608 (0.245)

Table S5.4 Summary Table for Macedonia

	Treatment as usual (N= 90)	Community mental care (N= 90)	Overall (N= 180)
**Age in years at baseline**			
Mean (SD)	46.5 (11.6)	47.7 (11.8)	47.1 (11.7)
**Female gender**			
Mean (SD)	0.544 (0.501)	0.633 (0.485)	0.589 (0.493)
**Living with/out a partner**			
Mean (SD)	0.0667 (0.251)	0.122 (0.329)	0.0944 (0.293)
**Employment status**			
Mean (SD)	0.367 (0.485)	0.467 (0.502)	0.417 (0.494)
**Above average income**			
Mean (SD)	0.0444 (0.207)	0.133 (0.342)	0.0889 (0.285)
**Education**			
Primary education	11 (12.2%)	6 (6.7%)	17 (9.4%)
Secondary education	41 (45.6%)	58 (64.4%)	99 (55.0%)
Lower vocational education	7 (7.8%)	3 (3.3%)	10 (5.6%)
Higher vocational and academic	31 (34.4%)	23 (25.6%)	54 (30.0%)
**ICD-10 psychotic spectrum disorders**			
Mean (SD)	0.289 (0.456)	0.189 (0.394)	0.239 (0.428)
**ICD-10 bipolar disorder**			
Mean (SD)	0.256 (0.439)	0.133 (0.342)	0.194 (0.397)
**ICD-10 major depressive disorder**			
Mean (SD)	0.267 (0.445)	0.511 (0.503)	0.389 (0.489)
**Long treatment history > 5 years**			
Mean (SD)	0.644 (0.481)	0.589 (0.495)	0.617 (0.488)
**WHODAS 2.0 at t1**			
Mean (SD)	39.0 (17.8)	31.3 (21.0)	35.1 (19.8)
**imputed Cognition – understanding & communicating**			
Mean (SD)	35.9 (20.6)	25.4 (24.0)	30.6 (22.9)
**imputed Mobility – moving & getting around**			
Mean (SD)	29.4 (25.6)	29.2 (28.2)	29.3 (26.8)
**imputed Self-care – hygiene, dressing, eating & staying alone**			
Mean (SD)	23.8 (23.8)	18.3 (26.2)	21.0 (25.1)
**imputed Getting along– interacting with other people**			
Mean (SD)	45.1 (25.2)	27.7 (28.0)	36.4 (27.9)
**imputed Life activities– domestic responsibilities, leisure, work & school**			
Mean (SD)	47.7 (23.6)	28.9 (28.7)	38.3 (27.8)

Table S5.4 Summary Table for Macedonia

	Treatment as usual (N= 90)	Community mental care (N= 90)	Overall (N= 180)
**imputed Participation– joining in community activities**			
Mean (SD)	48.6 (19.4)	44.5 (25.4)	46.6 (22.6)
**EQ-5D-3L utility at t1**			
Mean (SD)	0.654 (0.238)	0.590 (0.257)	0.622 (0.249)

Table S5.5 Summary Table for Montenegro

	Treatment as usual (N= 102)	Community mental care (N= 100)	Overall (N= 202)
**Age in years at baseline**			
Mean (SD)	50.1 (12.7)	49.3 (11.7)	49.7 (12.2)
**Female gender**			
Mean (SD)	0.382 (0.488)	0.500 (0.503)	0.441 (0.498)
**Living with/out a partner**			
Mean (SD)	0.186 (0.391)	0.110 (0.314)	0.149 (0.356)
**Employment status**			
Mean (SD)	0.157 (0.365)	0.220 (0.416)	0.188 (0.392)
**Above average income**			
Mean (SD)	0.0686 (0.254)	0.0200 (0.141)	0.0446 (0.207)
**Education**			
Primary education	15 (14.7%)	25 (25.0%)	40 (19.8%)
Secondary education	59 (57.8%)	48 (48.0%)	107 (53.0%)
Lower vocational education	16 (15.7%)	13 (13.0%)	29 (14.4%)
Higher vocational and academic	10 (9.8%)	13 (13.0%)	23 (11.4%)
**ICD-10 psychotic spectrum disorders**			
Mean (SD)	0.147 (0.356)	0.350 (0.479)	0.248 (0.433)
**ICD-10 bipolar disorder**			
Mean (SD)	0.108 (0.312)	0.0800 (0.273)	0.0941 (0.293)
**ICD-10 major depressive disorder**			
Mean (SD)	0.147 (0.356)	0.0700 (0.256)	0.109 (0.312)
**Long treatment history > 5 years**			
Mean (SD)	0.735 (0.443)	0.750 (0.435)	0.743 (0.438)
**WHODAS 2.0 at t1**			
Mean (SD)	25.5 (15.0)	21.3 (12.5)	23.4 (13.9)
**imputed Cognition – understanding & communicating**			
Mean (SD)	22.0 (25.1)	20.4 (24.3)	21.2 (24.6)
**imputed Mobility – moving & getting around**			
Mean (SD)	20.5 (27.3)	18.3 (26.2)	19.4 (26.7)
**imputed Self-care – hygiene, dressing, eating & staying alone**			
Mean (SD)	11.8 (19.1)	9.40 (15.6)	10.6 (17.4)
**imputed Getting along– interacting with other people**			
Mean (SD)	22.0 (20.0)	19.4 (14.9)	20.7 (17.7)
**imputed Life activities– domestic responsibilities, leisure, work & school**			
Mean (SD)	24.7 (24.5)	23.7 (22.9)	24.2 (23.7)

Table S5.5 Summary Table for Montenegro

	Treatment as usual (N= 102)	Community mental care (N= 100)	Overall (N= 202)
**imputed Participation– joining in community activities**			
Mean (SD)	42.8 (20.0)	36.5 (19.4)	39.6 (19.9)
**EQ-5D-3L utility at t1**			
Mean (SD)	0.654 (0.243)	0.718 (0.246)	0.685 (0.246)

Table S5.6 Summary Table for Romania

	Treatment as usual (N= 90)	Community mental care (N= 90)	Overall (N= 180)
**Age in years at baseline**			
Mean (SD)	53.5 (12.2)	53.2 (12.0)	53.4 (12.1)
**Female gender**			
Mean (SD)	0.556 (0.500)	0.544 (0.501)	0.550 (0.499)
**Living with/out a partner**			
Mean (SD)	0.0778 (0.269)	0.0889 (0.286)	0.0833 (0.277)
**Employment status**			
Mean (SD)	0.133 (0.342)	0.0556 (0.230)	0.0944 (0.293)
**Above average income**			
Mean (SD)	0 (0)	0.0111 (0.105)	0.00556 (0.0745)
**Education**			
Primary education	32 (35.6%)	41 (45.6%)	73 (40.6%)
Secondary education	23 (25.6%)	27 (30.0%)	50 (27.8%)
Lower vocational education	22 (24.4%)	11 (12.2%)	33 (18.3%)
Higher vocational and academic	13 (14.4%)	11 (12.2%)	24 (13.3%)
**ICD-10 psychotic spectrum disorders**			
Mean (SD)	0.389 (0.490)	0.489 (0.503)	0.439 (0.498)
**ICD-10 bipolar disorder**			
Mean (SD)	0.0667 (0.251)	0.0778 (0.269)	0.0722 (0.260)
**ICD-10 major depressive disorder**			
Mean (SD)	0.378 (0.488)	0.333 (0.474)	0.356 (0.480)
**Long treatment history > 5 years**			
Mean (SD)	0.633 (0.485)	0.722 (0.450)	0.678 (0.469)
**WHODAS 2.0 at t1**			
Mean (SD)	36.7 (16.8)	37.2 (15.5)	37.0 (16.1)
**imputed Cognition – understanding & communicating**			
Mean (SD)	32.7 (19.7)	34.9 (18.7)	33.8 (19.2)
**imputed Mobility – moving & getting around**			
Mean (SD)	24.7 (22.7)	22.6 (21.4)	23.7 (22.0)
**imputed Self-care – hygiene, dressing, eating & staying alone**			
Mean (SD)	22.2 (23.7)	20.7 (21.7)	21.4 (22.7)
**imputed Getting along– interacting with other people**			
Mean (SD)	45.3 (23.1)	46.8 (20.3)	46.0 (21.7)

Table S5.6 Summary Table for Romania

	Treatment as usual (N= 90)	Community mental care (N= 90)	Overall (N= 180)
**imputed Life activities– domestic responsibilities, leisure, work & school**			
Mean (SD)	32.0 (21.7)	32.7 (18.5)	32.3 (20.1)
**imputed Participation– joining in community activities**			
Mean (SD)	53.1 (16.2)	53.9 (18.2)	53.5 (17.2)
**EQ-5D-3L utility at t1**			
Mean (SD)	0.733 (0.172)	0.738 (0.187)	0.735 (0.179)
