# Supplementary material for: Community versus institutionalised care for people with severe mental illness in five countries in Southeast Europe: pooled analysis of five randomised trials
Source: BMJ Glob Health. 2025 Oct 23;10(10):e018594. doi: 10.1136/bmjgh-2024-018594 (PMC12551481; doi:10.1136/bmjgh-2024-018594)
Supplement: online supplemental file 6 [file bmjgh-10-10-s006.pdf]

Supplemental Material 6

Table S6.1: Predicted marginal means for changes in WHODAS disability by treatment condition over time (corresponding to figure 2 left side)

Months#Condition	Margin	Delta-method Std. Err.	z
1#TAU	35.27851	0.631121	55.9
1#CMHC	34.92192	0.633159	55.16
12#TAU	30.09538	0.705535	42.66
12#CMHC	28.86198	0.700196	41.22
18#TAU	33.53946	0.703673	47.66
18#CMHC	28.63126	0.695783	41.15

Table S6.2: Predicted marginal means for changes in WHODAS disability by treatment condition by country over time (corresponding to figure 2 right side)

Months#Country#Condition	Margin	Delta-method Std. Err.	z
1#Bulgara#TAU	41.15972	0.875138	47.03
1#Bulgara#CMHC	40.80314	0.873577	46.71
1#Croatia#TAU	37.59064	0.921198	40.81
1#Croatia#CMHC	37.23406	0.92568	40.22
1#Macedonia#TAU	34.64966	0.917833	37.75
1#Macedonia#CMHC	34.29308	0.918719	37.33
1#Montenegro#TAU	26.60454	0.882171	30.16
1#Montenegro#CMHC	26.24795	0.885118	29.65
1#Romania#TAU	36.93595	0.903258	40.89
1#Romania#CMHC	36.57936	0.903874	40.47
12#Bulgara#TAU	35.56712	0.922305	38.56
12#Bulgara#CMHC	34.33372	0.915926	37.49
12#Croatia#TAU	32.37015	0.968653	33.42
12#Croatia#CMHC	31.13675	0.965544	32.25
12#Macedonia#TAU	28.38095	0.977066	29.05
12#Macedonia#CMHC	27.14755	0.971353	27.95
12#Montenegro#TAU	21.26782	0.94139	22.59
12#Montenegro#CMHC	20.03442	0.936144	21.4
12#Romania#TAU	32.51447	0.958939	33.91
12#Romania#CMHC	31.28107	0.960583	32.56
18#Bulgara#TAU	39.20356	0.923378	42.46
18#Bulgara#CMHC	34.29536	0.916481	37.42
18#Croatia#TAU	35.3814	0.972238	36.39
18#Croatia#CMHC	30.4732	0.966397	31.53
18#Macedonia#TAU	31.69413	0.981604	32.29
18#Macedonia#CMHC	26.78594	0.974408	27.49
18#Montenegro#TAU	25.04216	0.949381	26.38

18#Montenegro#CMHC	20.13396	0.942607	21.36
18#Romania#TAU	34.55467	0.936234	36.91
18#Romania#CMHC	29.64647	0.933195	31.77

Table S6.3: Predicted marginal means for changes in EQ-5D utilities by treatment condition over time (corresponding to figure 3 left side)

Months#Condition	Margin	Delta-method Std. Err.	z
1#TAU	0.666013	0.007465	89.22
1#CMHC	0.666593	0.007489	89.01
12#TAU	0.701851	0.008023	87.49
12#CMHC	0.709924	0.007966	89.12
18#TAU	0.669536	0.008051	83.16
18#CMHC	0.735261	0.007985	92.08

Table S6.4: Predicted marginal means for responder rate by treatment condition over time (corresponding to figure 3 right side)

Months#Condition	Margin	Delta-method Std. Err.	z
1#TAU	2.94E-05	0.017805	0
1#CMHC	-3E-05	0.017863	0
12#TAU	0.458103	0.019874	23.05
12#CMHC	0.496948	0.019722	25.2
18#TAU	0.364047	0.019823	18.37
18#CMHC	0.458333	0.019605	23.38
