# Supplementary material for: Community versus institutionalised care for people with severe mental illness in five countries in Southeast Europe: pooled analysis of five randomised trials
Source: BMJ Glob Health. 2025 Oct 23;10(10):e018594. doi: 10.1136/bmjgh-2024-018594 (PMC12551481; doi:10.1136/bmjgh-2024-018594)
Supplement: online supplemental file 7 [file bmjgh-10-10-s007.pdf]

Supplemental Material 7

Table S7.1: Base-case and sensitivity analyses of CMH vs TAU on outcome at t_{18}

Analysis						95% CI	
.	Outcome	*b*	*SE*	*z*	*p*	lower	upper
Base-case							
.	Disability	–4.55	1.21	–3.75	<0.001	–6.93	–2.17
.	Response	0.06	0.03	1.94	0.052	–0.00	0.13
.	Quality of Life	0.07	0.014	4.56	<0.001	0.04	0.09
Sensitivity							
.	Disability	–4.97	1.24	–4.02	<0.001	–7.40	–2.55
.	Response	0.09	0.04	2.52	0.012	0.02	0.16
.	Quality of Life	0.06	0.01	4.36	<0.001	0.04	0.09
